# A narrow host-range and lack of persistence in two non-target insect species of a bacterial symbiont exploited to deliver insecticidal RNAi in Western Flower Thrips

**DOI:** 10.3389/finsc.2023.1093970

**Published:** 2023-03-08

**Authors:** Miranda M. A. Whitten, Qi Xue, Clauvis Nji Tizi Taning, Reuben James, Guy Smagghe, Ricardo del Sol, Matthew Hitchings, Paul Dyson

**Affiliations:** ^1^ Institute of Life Science, Swansea University Medical School, Singleton Park, Swansea, United Kingdom; ^2^ Department of Plants and Crops, Faculty of Bioscience Engineering, Ghent University, Ghent, Belgium

**Keywords:** symbiotic bacteria, host-range, Western flower Thrips, European bumblebee, pirate bug

## Abstract

**Introduction:**

Insecticidal RNAi is a targeted pest insect population control measure. The specificity of insecticidal RNAi can theoretically be enhanced by using symbiotic bacteria with a narrow host range to deliver RNAi, an approach termed symbiont-mediated RNAi (SMR), a technology we have previously demonstrated in the globally-invasive pest species Western Flower Thrips (WFT).

**Methods:**

Here we examine distribution of the two predominant bacterial symbionts of WFT, BFo1 and BFo2, among genome-sequenced insects. Moreover, we have challenged two non-target insect species with both bacterial species, namely the pollinating European bumblebee, Bombus terrestris, and an insect predator of WFT, the pirate bug Orius laevigatus.

**Results:**

Our data indicate a very limited distribution of either symbiont among insects other than WFT. Moreover, whereas BFo1 could establish itself in both bees and pirate bugs, albeit with no significant effects on insect fitness, BFo2 was unable to persist in either species.

**Discussion:**

In terms of biosafety, these data, together with its more specific growth requirements, vindicate the choice of BFo2 for delivery of RNAi and precision pest management of WFT.

## 1 Introduction

Insecticides are extensively used worldwide as part of modern agricultural practice and to limit vector-borne diseases. Of the 3 million tons of pesticides used annually, insecticides account for about 30% of this total and the bulk of these are synthetic chemicals (1). Unfortunately, pest resistance to conventional insecticides is commonplace and they also have well-documented toxic impacts on non-target beneficial insect species (2). Moreover, certain compounds can bioaccumulate and have toxic effects on other invertebrates and vertebrate animals higher in the food-chain.

Although making up a much smaller proportion of the global insecticide market, biopesticides are generally considered as having fewer deleterious effects on the environment. Examples are microbials such *Bacillus thuringiensis*, the insecticidal Bt toxins these produce, and botanicals such as pyrethrum. However, in addition to issues with pest insect resistance, many biopesticides can have either lethal or negative sublethal effects on pollinators and beneficial arthropods such as parasitoids and predators (3).

These limitations highlight the need to develop alternative, more targeted pest insect management strategies. A significant step in this direction is the use of insecticidal gene silencing by RNA interference (RNAi), which is expected to revolutionize pest control (4). RNAi can be lethal to an insect if an essential gene is targeted or can reduce the population size if a gene influencing fertility is silenced. A level of targeted specificity against a given pest is achieved by the design of the interfering RNA so that it exactly matches only the mRNA transcript of the pest gene, and not similar genes of non-target species. However, this design specificity is to some extent constrained due to the conservation of essential gene sequences and the fact that insect RNAi is typically achieved by delivery of long dsRNA which is ‘diced’ up *in insecta* into many different siRNAs, some of which could in theory act on mRNAs with complementary sequences in non-target species (5). Nevertheless, in June 2017, the U.S. Environmental Protection Agency approved the first *in planta* RNAi product against insects for commercial use. This genetically modified maize expresses a dsRNA targeting the Western Corn Rootworm (WCR) *snf7* gene in combination with Bt proteins and is being introduced for commercial use. An ecological risk assessment for this RNAi approach targeting the WCR *snf7* gene did not reveal any adverse effects on a variety of non-target insect species (6).

The efficiency of insecticidal RNAi can vary depending on the insect species and the method of delivering dsRNA (7). We have been investigating insecticidal RNAi in *Frankliniella occidentalis*, Western Flower Thrips (WFT), a globally invasive polyphagous insect pest and a vector of plant-pathogenic tospoviruses (8). WFT resistance to several conventional chemical insecticides is well documented (9). Experimental RNAi in WFT has been achieved by micro-injection of dsRNA (10), but strategies that can be scaled up for field applications depend on insects acquiring interfering RNA in their diets (11). However, due to the activity of RNases in the upper digestive tract, many insect species can evade the lethal effects of ingested dsRNA, as is the case for WFT (12). The use of symbiotic bacteria to deliver dsRNA represents a possible alternative (13, 14), whereby the dsRNA is synthesized *in insecta* by bacteria that colonize the gastrointestinal (GI) tract. This approach is termed symbiont-mediated RNAi (SMR). As an insecticide, SMR potentially offers a second tier of specificity if the symbiotic bacteria fail to colonize other non-target insect species. Here, we assess the specificity and persistence of the two dominant WFT symbionts named Bacteria of *F. occidentalis* 1 and 2, BFo1 and BFo2, found in geographically isolated WFT populations (15–17). BFo1 is related to *Erwinia*, whereas BFo2 has some similarities to *Pantoea* (15). Analysis of the reference WFT hologenome indicates that BFo1 can account for 72% and BFo2 15% of the WFT gut microbiome (18). Here, we characterize their distribution in other insects by analysis of genomic data, and we examine the growth requirements of these bacteria and their ability to colonize both a non-target pollinator, the European bumblebee *Bombus terrestris*, and an insect predator currently used for bio-control of WFT, the pirate bug *Orius laevigatus*. Taken together, these analyses provide evidence for the narrow host specificity of BFo2. This is first study of this type and it vindicates the choice of BFo2 for delivery of insecticidal RNAi for precision control of WFT by SMR.

## 2 Materials and methods

### 2.1 Bacterial strains, growth conditions and bacterial identification

The BFo bacterial strains employed in this study are listed in **Table 1**. To measure growth, a M9-based minimal medium was used containing 0.25M Na_2_HPO_4_.7H_2_O, 0.1M KH_2_PO_4_, 2mM MgSO_4_ and 100uM CaCl_2_, pH7.2. The final concentration of either glucose or sucrose as C source was 0.4%, and either 0.1 M NH_4_Cl or 40 mM L-asparagine was added as N source. NaCl, at a final concentration of 40 mM, was added as an osmolyte. To measure growth, starting cultures contained 10^2^ cells per ml in 200 ul of medium in the wells of microplates. Growth curves were calculated, measuring turbidity at OD_600_ for triplicate cultures grown at 30°C. Doubling times were calculated for the period of exponential growth (19).

**Table 1:** Bacterial strains used.

Bacterial strain	Antibiotic resistance	Reference
BFo1	-	(15)
BFo1 + pdag-GFP	Ampicillin	(18)
BFo1 + pMK	Kanamycin	(18)
BFo2	-	(15)
BFo2 + pdag-GFP	Ampicillin	(18)

To prepare bacteria for insect feeding, the strains were grown in liquid LB medium at 30°C, as previously described (18). Bacteria were recovered from insects by plating on LB agar containing, as appropriate, either 50 ug ml^-1^ kanamycin or 100 ug ml^-1^ ampicillin. Where stated, the identity of colonies was verified by colony PCR, using primers specific for either BFo1 or BFo2, as previously described (18).

### 2.2 Exploring BFo1 and BFo2 host range in the sequence read archive database

The results of the Sequence Taxonomic Analysis Tool (STAT) (20) for each Sequence Read Archive publicly available submission were queried using the Google BigQuery user interface. The SQL queries were composed to search all metadata and taxonomic analysis results that contained at least a single k-mer for the corresponding NCBI taxonomy IDs; 1628855 (BFo1) and 1628856 (BFo2). The following fields were selected *m.acc, m.sample_acc, m.biosample, m.sra_study, m.bioproject, total_count, organism, tax_id, rank, name, self_count*. A further manual consolidation of this list was applied to limit results to only those which were associated with an insect host.

### 2.3 Bumblebees

Bumblebee workers (*B. terrestris*) used in our experiments were bought from the company BioBest (Westerlo, Belgium) and were reared at 30°C, 65% relative humidity and 24 h darkness in climate-controlled incubators. A 50% sucrose solution (BioGluc, Biobest) and pollen (Apihurdes, Spain) were provided as the food source to the bumblebees *ad libitum*.

**Figure 1:**
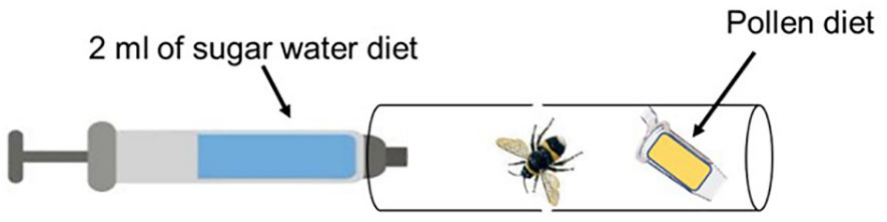
Bumblebee feeding tube. For treatment groups, BFo bacteria were added to the sugar water solution. The sugar water solution was replaced every 24 h.

Prior to administering the bacteria (BFo1 and BFo2), bumblebees were individually placed in single housing tubes (**Figure 1**) and starved for 3 h without any food. Then, each bumblebee was provided with pollen and either 2 ml of sucrose diet containing the same bacterial density (1.27 x10^5^ cells/ml) of BFo1 for 48 h (40 bumblebees and 2 replications) or BFo2 for 72 h (60 bumblebees and 3 replications). The diet was renewed daily to ensure that the bumblebees would feed on live bacteria. For the control groups, the same number of bumblebees as in the treatment groups (BFo1 and BFo2) were provided with pollen and 2 ml of 50% sucrose solution that was bacteria-free. After the exposure period, the bumblebees were transferred to new cages containing fresh sucrose and pollen diet. The diet was refreshed daily for 7 days, during which daily food intake by each bumblebee was evaluated by weighing the tubes loaded with pollen and sugar water before and after feeding. The numbers of dead bumblebees were also recorded daily during this period.

To evaluate whether BFo1 and BFo2 could successfully colonize the bumblebee gut, 3 bumblebees were randomly collected at day 1, 2 and 5 post exposure to either BFo1 and BFo2 for gut extraction. In brief, the entire gut of 3 bumblebees were extracted under sterile conditions and immediately homogenized in 300 μl of sterile phosphate buffered saline (PBS). After centrifugation at 2000 rpm for 10 min, 200 μl of the supernatant was plated on Lysogeny broth (LB) agar plates and incubated at 29°C overnight (maximum 24 h). Single colonies of the engineered bacteria (BFo1 and BFo2) expressing the green fluorescent protein (GFP) were detected using a fluorescence microscope.

### 2.4 Pirate bugs

*O. laevigatus* and their supplementary food source, Nutrimac™, were obtained from Dragonfli Ltd (Essex, UK). The pirate bugs were reared in Bugdorms (Megaview Science Co. Ltd, Taiwan) on chrysanthemum plants and runner bean seedlings, with Nutrimac™ *ad libitum*.

For membrane feeding we established an artificial diet of tryptic soy broth plus yeast extract (TSBY (12);) into which was added, for the treatment groups, 10^7^ ml^-1^ of either BFo1 containing plasmid pMK, conferring stable resistance to kanamycin (18), or BFo2 with the GFP plasmid. These solutions were then sealed inside the lids of microfuge tubes using stretched Parafilm^®^ M film as a membrane. Control feeding solutions consisted of TSBY only. The pirate bug diet was also supplemented throughout the experiments with Nutrimac™, and a piece of folded tissue as a refuge. Triplicate groups of 12 adult pirate bugs were placed in glass bijou bottles, together with the bacterial feeding solution. Pirate bugs were not starved prior to membrane feeding as this was found to negatively impact their survival. The pirate bugs were exposed to this diet *via* membrane feeding for 6 days and the diet was renewed daily. At day 7, to assess uptake of the bacteria, we sampled individual insect cuticles, whole homogenized surface-sterilized insects, and frass samples. Pirate bug cuticles were washed with sterile water to recover the bacteria. Subsequently, the insects were surface sterilized by immersion in iodine tincture for 30 seconds then rinsed with water. Insects were then homogenized in 100 μl H_2_O using a micropestle and individual homogenates were plated on LB agar. Frass samples were obtained by swabbing random excreted deposits on the surfaces within the bijou bottle.

We also assessed persistence of BFo bacteria in *O. laevigatus* adults following membrane feeding, by repeating the above protocol but maintaining the insects for an additional 3 days with a diet lacking bacteria.

### 2.5 Pirate bug predation on colonized WFT

To study pirate bug uptake of the symbiotic bacteria from predated WFT, we first established a prey WFT population harboring both BFo1 with plasmid pMK and BFo2 with pdag-GFP, as previously described (18). Blue tracker dye was used in the WFT diet to ensure all prey individuals had ingested both BFo1 and BFo2, and randomly selected prey WFT were tested to confirm the presence of the bacteria by culturing whole body homogenates followed by PCR.

Forty-two *O. laevigatus* (equal numbers of adult males and females and final-stage nymphs) were then distributed between 6 glass bijou bottles each containing approximately 40 prey WFTs. Predation was allowed for 3 days, during which all the WFTs were predated. The pirate bugs were then maintained without prey for a further 10 days on fresh TSBY and Nutrimac™ as food supplements (renewed every 1-2 days), plus leaves from laboratory-germinated bean seedlings as a substrate for oviposition. Tissue paper refuges were provided at all times. The F_0_ and subsequent F_1_ generation pirate bugs were analyzed for the presence of both BFo bacteria in surface-sterilized whole insect homogenates, cuticle swabs and in frass at 0, 3 and 7 days after the cessation of predation. Pirate bug survival was also noted. Control insects were fed as above but using WFT free from engineered BFo2.

### 2.6 Statistical analyses

Data distribution was verified using the Shapiro-Wilk test. For data with a normal distribution, an unpaired t-test was performed to evaluate if there was significance difference (p < 0.05) between the control and the test groups. For data that was not normally distributed, a Mann-Whitney U-test (p < 0.05) was performed. Analyses were performed using JMP software (SAS Institute Inc.) and Prism v. 6 (GraphPad Software, San Diego, www.graphpad.com).

## 3 Results

### 3.1 Growth requirements of BFo1 and BFo2

The growth requirements of the two WFT symbionts were compared by deriving a minimal medium that supported their growth and then comparing growth rates with different carbon and nitrogen sources, and in the presence or absence of NaCl as an osmolyte. Firstly, it was noted that the doubling time in all media for BFo1 was appreciably faster than for BFo2 (**Table 2**). In addition, the analysis indicated that for all three variables, BFo2 was the more fastidious bacterium. For this species, optimal growth was achieved with sucrose rather than glucose as the C-source, and with an amino acid such as asparagine rather than ammonium as the N-source. BFo1, on the other hand, grew equally well with both the C- and N-sources tested. In addition, inclusion of 40 mM NaCl as an osmolyte was required for optimal growth of BFo2, whereas there was no similar requirement for BFo1. Given these requirements for rapid growth, we could conclude that, of the two species, BFo2 is less likely to compete as a free-living bacterium in the environment.

**Table 2:** Doubling times (min) for the two WFT symbiotic bacteria, BFo1 and BFo2, grown in supplemented minimal medium.

C source	N source	osmolyte	BFo1	BFo2
Glucose	Ammonium	N	43	104
Glucose	Asparagine	N	43	94
Glucose	Ammonium	Y	45	90
Glucose	Asparagine	Y	43	76
Sucrose	Ammonium	N	44	95
Sucrose	Asparagine	N	42	70
Sucrose	Ammonium	Y	47	75
Sucrose	Asparagine	Y	46	59

### 3.2 Identification of BFo1 and BFo2 in insect DNA sequence libraries

The host range of BFo1 and BFo2 was explored by interrogating the taxonomic distribution of sequence reads in Sequence Read Archive (SRA) entries using the Sequence Taxonomic Analysis Tool (STAT). Insect-derived SRA entries containing at least one unique k-*mer* from BFo1 or BFo2 genome sequences were identified, and the count of BFo1 or BFo2 unique k-*mers* retrieved for each SRA experiment, alongside BioSample and BioProject identifiers. A total of 121,911 insect-derived SRA experiments were analysed, revealing 269 SRA experiments containing BFo2 (0.22%) and 299 SRA entries containing BFo1 (0.25%) with DNA as source material. Only 13 SRA experiments contained both BFo1 and BFo2 unique k-mers (**Table 3**) within the same SRA experiment. Since multiple SRA experiments are replicates from the same BioSample, the total number of unique BioSamples containing BFo1 or BFo2 was determined. Out of a total 43,122 insect BioSamples with SRA data, only 207 contained BFo1 (0.48%), 167 contained BFo2 (0.39%), and 9 contained both. The total count, SRA experiments, k-*mers* and BioSamples containing BFo1 or BFo2 per species is shown in **Supplementary Table 1**.

**Table 3:** Number of insect derived SRA experiments and BioSamples showing association to BFo1 andor BFo2 unique k-mers.

	SRA experiments	Number of biosamples
**Total data set**	121911	43122
**Bfo1**	299	207
**Bfo2**	269	167
**Bfo1 and Bfo2**	13	9

As expected, *F. occidentalis* contains the highest number of unique BFo1 k-*mers*, with BFo2 k-*mers* being the second most abundant originating from a single BioSample. There is a strong bias towards the presence of *Anopheles* sp. in the dataset (105 BioSamples with BFo1, 55 with BFo2), although this is probably a consequence of the genus being extensively studied due to the relevance of mosquitos as disease vectors. Further, these numbers constitute only small fractions of the total 37,793 *Anopheles* sp. BioSamples with SRA data from a DNA source, which means BFo1 and BFo2 are found in only 0.28% and 0.15% of *Anopheles* sp. BioSamples respectively.

The presence of BFo1 seems rare in the genus *Apis* (with only 5 BioSamples), although BFo2 is a more common occurrence with 32 BioSamples showing association to this genus (**Supplementary Table 1**). *Apis mellifera* showed the third most abundant BFo2 k-mer count after *Anopheles gambiae* and *F. occidentalis*. However, the latter observation must also be considered within the context of 24,955 SRA experiments on *Apis* sp. subjects, across 20,037 BioSamples, which indicates that *Apis* sp.-Bfo2 association may be a transient, isolated event rather than a widespread occurrence. Similarly, *Danaus plexippus* (Monarch butterfly) is well represented with high k-*mer* counts and 10 and 19 BioSamples containing BFo1 and BFo2, respectively.

The main observation derived from these analyses is that, despite a very lenient threshold used (one unique k-mer or more), there is a very limited number of insect BioSamples whose corresponding SRA data indicate the presence of BFo1 or BFo2. A total of 57 insect species showed the presence of BFo1, and 51 with BFo2, with most of these represented by a single BioSample and very low k-*mer* counts indicating the presence of these bacteria to be either transient or sporadically present rather than having a conserved biological association (**Supplementary Table 1**).

### 3.3 Persistence and fitness effects of BFo1 bacteria in bumblebees

A strain of BFo1 expressing GFP was used to assess whether the bacteria could colonize the bumblebee gut as this provided a facile means to discriminate between members of the resident endogenous microbiome and the introduced bacteria. We previously demonstrated that the GFP plasmid, pdag-GFP, is stably inherited in BFo1 over a 10-day period, approximately 100 generations, in the absence of antibiotic selection (18). For the test group, 10^5^ bacteria were added to a feeding solution containing 50% sucrose. Prior to bee feeding, we established that the bacteria were viable in this solution for at least 24 h by monitoring green fluorescence under the microscope. Bees were also provided a separate source of pollen. In the absence of either nutrient source, bees died within 24 h, and in the presence of only pollen, they died within 48 h. Consequently, we could be confident that all bees in the treatment group ingested the bacteria in the 48 h period in which they were exposed to the feeding solution. A control group was exposed to bacteria-free 50% sucrose feeding solution together with pollen.

For a 5-day period thereafter (i.e. day 3 to day 8) both test and control groups of bees were provided with a bacteria-free feeding solution and pollen. At days 4, 5 and 8, three bees from each group were taken for gut dissection, gut homogenization and subsequent plating of gut bacteria. GFP-expressing BFo1 were detected at each time point for the test group, but not from the control group. Indeed, the number of GFP-expressing BFo1 bacteria recovered from gut homogenates increased approximately 100-fold between days 3 and 8, indicative of successful colonization in this period (**Supplementary Figure 1**).

**Figure 2:**
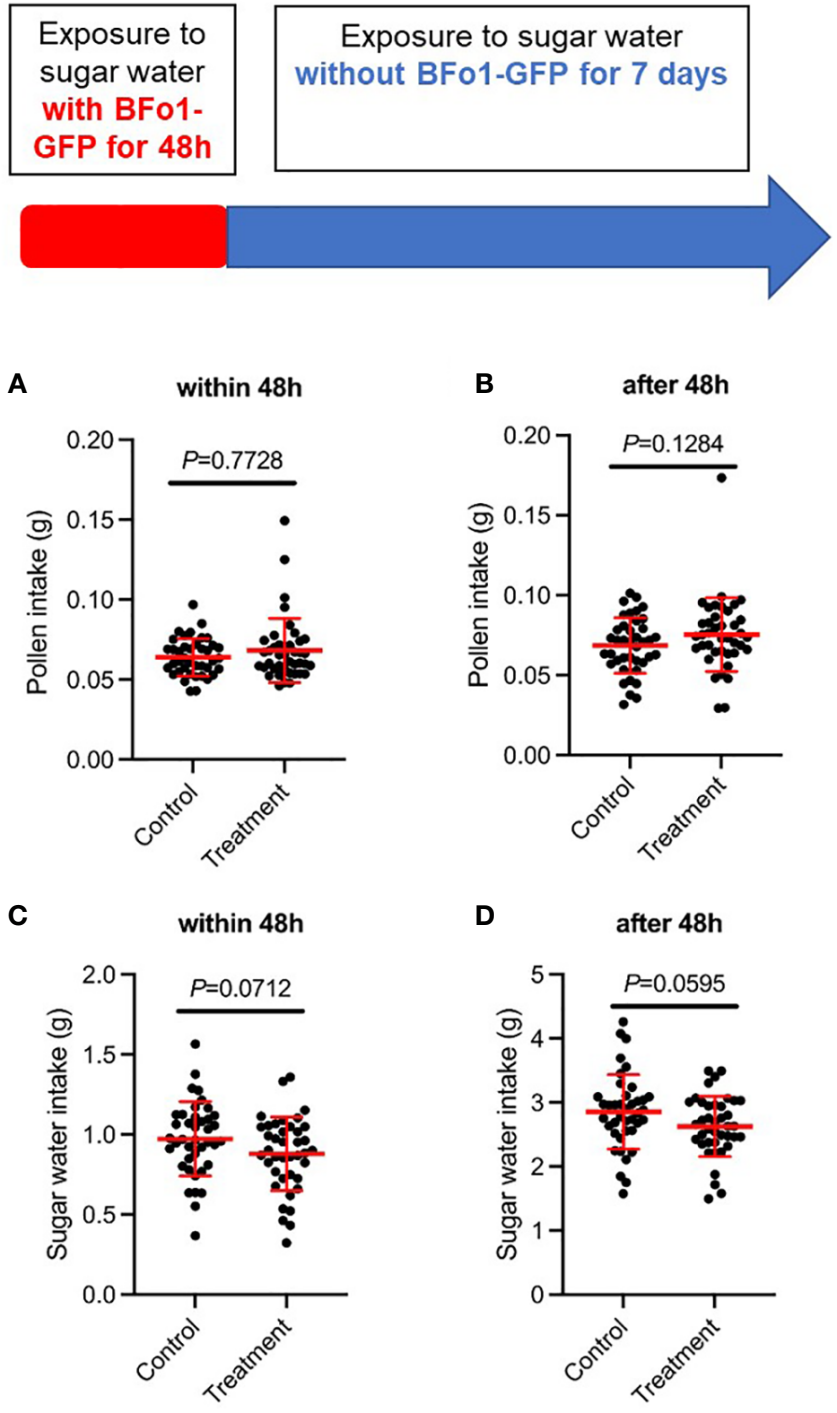
Ingestion of BFo1 has minor effects on bumblebee feeding behavior. Both pollen **(A, B)** and sugar water **(C, D)** intake were assessed for the initial 48 h bacterial exposure time **(A, C)**, and for the subsequent 5 days after withdrawal of the bacteria **(B, D)**.

To assess any effects of BFo1 colonization on the fitness of the bees, we measured food intake (sucrose solution and pollen) and mortality for 8 days after the ingestion of the bacteria. For both the initial 48 h bacterial exposure period, and the subsequent 5-day period, there were minor, but statistically insignificant, differences in feeding behavior between control and test groups (**Figure 2**). The test group tended to ingest slightly more pollen and less sucrose solution than the control group. However, there were no differences in mortality between the test and control groups during this period.

### 3.4 Persistence and fitness effects of BFo2 bacteria in bumblebees

To test colonization of bees by BFo2, we again utilized a strain containing a stable plasmid to express GFP (18). A similar experimental set-up as for the BFo1 experiments was used, except that the initial exposure to the bacteria in the sugar water was for 72 h (the longer exposure period was selected as initial studies revealed no evidence for colonization after 48 h). The sugar water was inoculated with 10^5^ bacteria, and we observed no loss of bacterial viability in this solution. After replacing this with sterile sugar water from day 4 onwards, triplicate insects were sacrificed at days 5, 6 and 8 and their guts dissected out, homogenized and plated. However, we were unable to detect any BFo2 colonies in these gut homogenates, indicating this bacterium is unable to survive in the bee gut.

**Figure 3:**
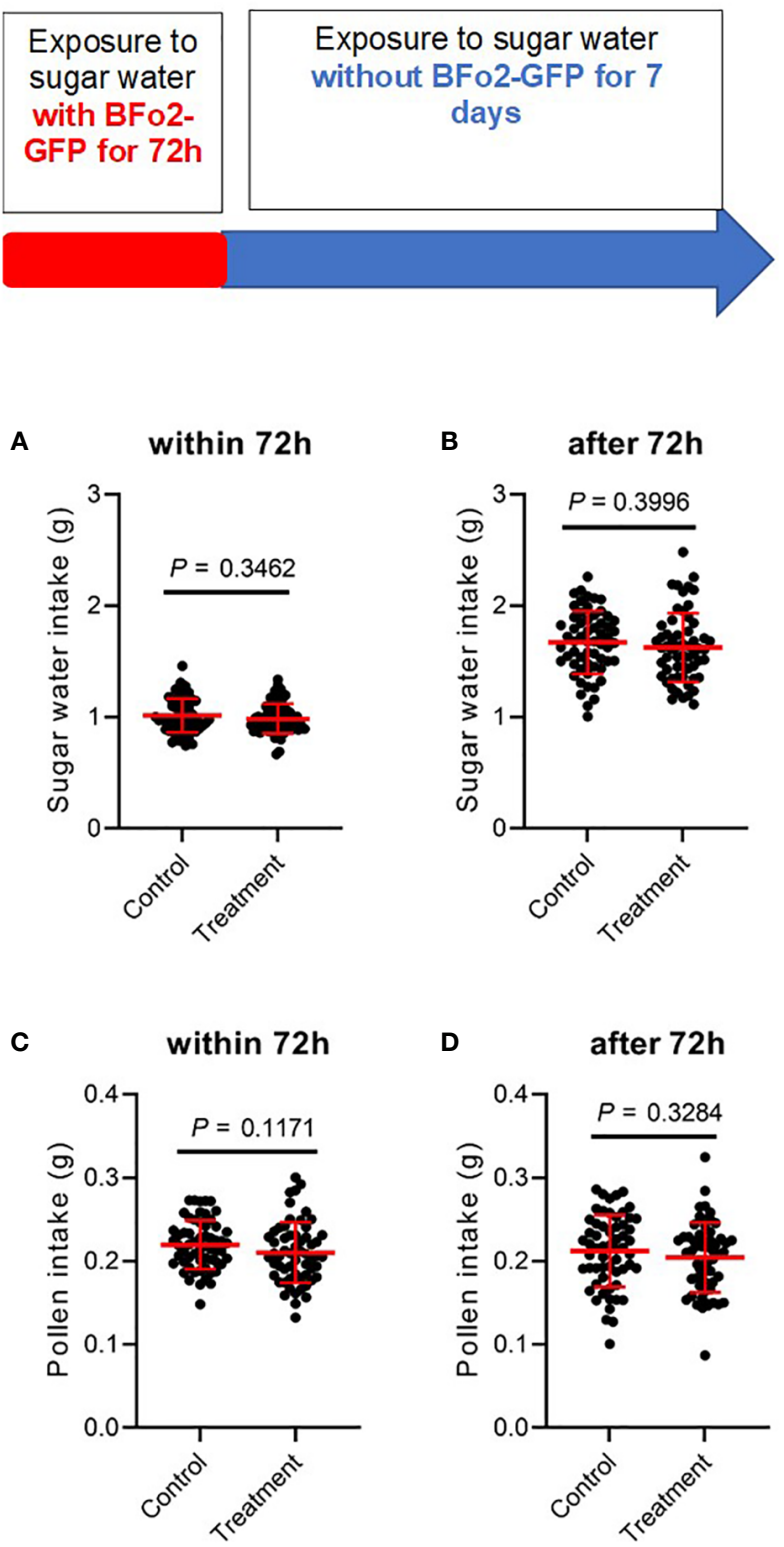
Ingestion of BFo2 has negligible effects on bumblebee feeding behavior. Both pollen **(A, B)** and sugar water **(C, D)** intake were assessed for the initial 72 h bacterial exposure time **(A, C)**, and for the subsequent 7 days after withdrawal of the bacteria **(B, D)**.

Fitness effects were assessed by measuring food intake, namely sucrose solution and pollen (**Figure 3**), and mortality over a 10-day period covering the initial 3-day exposure to the bacteria and the subsequent 7 days after switching the diet to sterile sugar water. No significant differences were observed between the control and treatment groups over this period for either feeding or mortality.

### 3.5 Survival and persistence of BFo bacteria introduced individually *via* membrane feeding in pirate bugs

Commercial *O. laevigatus* that had not been previously exposed to WFT were used for these analyses. Prior to assessing whether either bacterial species could colonize pirate bugs, we homogenized 2 pools of 10 insects, and also their artificial food source, Nutrimac™ – a mixture of the eggs of *Ephestia kuehniella* and semolina, then plated the homogenates on LB growth medium. Culturable bacteria were pooled and subjected to PCR reactions with primer pairs specific for BFo1 and BFo2 (18). No amplicons were detected, indicating the absence of these bacteria in both the insects and their food source.

The artificial TSBY diet was inoculated with 10^7^ ml^-1^ of either BFo1 containing plasmid pMK, conferring stable resistance to kanamycin (18), or BFo2 with the GFP plasmid. Adult pirate bugs were exposed to this diet *via* membrane feeding for 6 days. At day 7, to assess uptake of the bacteria, we sampled insect cuticles, whole homogenized surface-sterilized insects, and frass samples. We observed that in the absence of TSBY under experimental conditions, the survival of *O. laevigatus* was extremely poor over 3 days and thus we could be confident that vast majority of membrane-fed pirate bugs had ingested BFo bacteria during the experiment.

**Figure 4:**
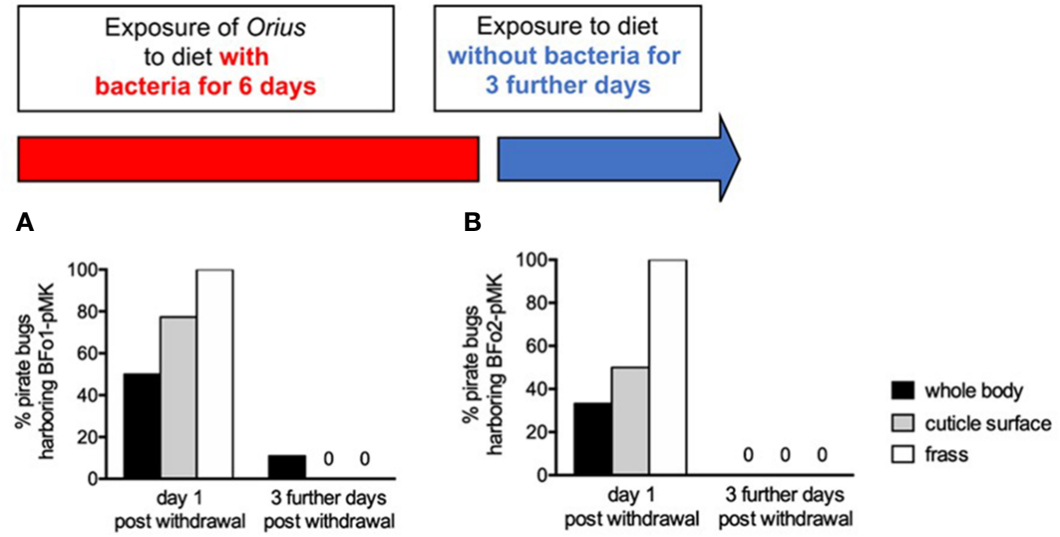
Persistence of BFo1 and BFo2 in pirate bugs *O. laevigatus* after forced membrane feeding. The persistence of BFo1 **(A)** and BFo2 **(B)** was evaluated by assessing the proportion of insects harboring the bacteria in their frass, on their cuticles and, after surface sterilization, within homogenates of whole bodies.

For BFo1 treated insects, these samples were plated on LB medium containing kanamycin. All kanamycin-resistant colonies derived from whole surface-sterilized insect homogenates or frass proved to be BFo1, as were the majority of colonies (>95%) from swabbed insect cuticles as verified by colony PCR. Half of the individual insect homogenates yielded BFo1, as did approximately three-quarters of cuticle swabs (77.3%) and all frass samples. We repeated this analysis 3 days after withdrawal of the bacteria from the diet, but at this time point only 11% of insect homogenates yielded BFo1, and the bacteria were not detected in cuticle swabs or frass samples (**Figure 4**).

For the treatment group exposed to BFo2 in their diet, samples were similarly obtained and analyzed, revealing that at day 7 one third of homogenized surface-sterilized insects yielded the bacteria, as did one half of cuticle swabs and all frass samples. However, 3 days after withdrawal from the diet, BFo2 bacteria were not detected in any samples (**Figure 4**).

### 3.6 Survival and persistence of BFo bacteria in pirate bugs predating on WFT

**Figure 5:**
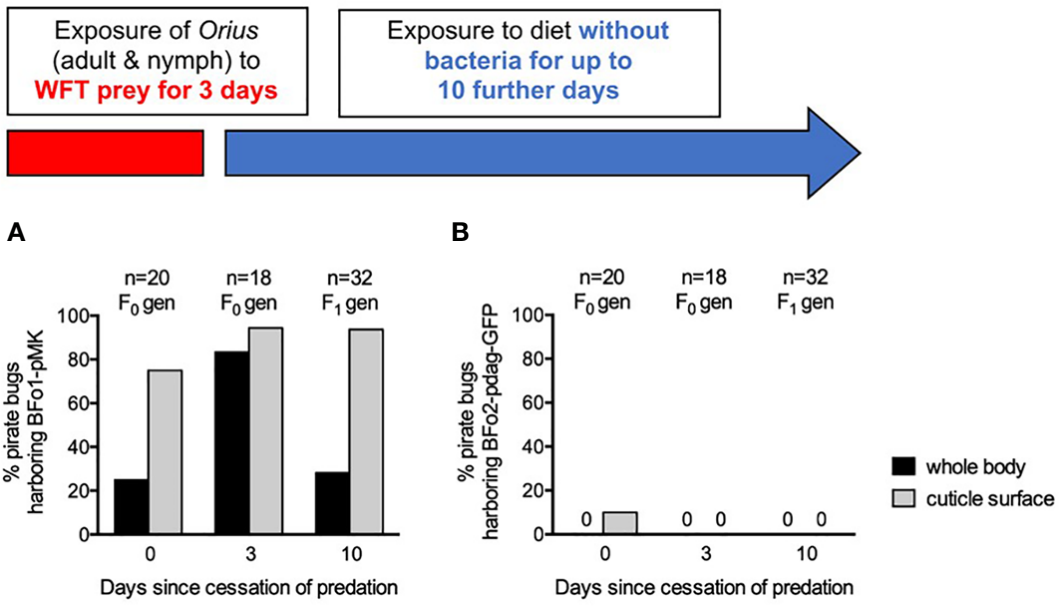
Persistence of BFo1 and BFo2 in pirate bugs *O. laevigatus* after WFT predation. The persistence of BFo1 **(A)** and BFo2 **(B)** was evaluated by assessing the proportion of insects harboring the bacteria on their cuticles and, after surface sterilization, within homogenates of whole bodies.

A WFT population harboring both BFo1 with plasmid pMK and BFo2 with pdag-GFP, as previously described (18), was fed to pirate bug adults and nymphs over a period of 3 days, prior to transferring them to an artificial diet for a further 10 days. F_0_ and subsequent F_1_ generation pirate bugs were analyzed for the presence of both bacteria in surface-sterilized whole insect homogenates, cuticle swabs and in frass at day 0, day 3 and day 10 after cessation of predation (**Figure 5**).

At day 0 after cessation of predation, homogenates of 25% of insects yielded BFo1, but BFo2 was not detected in any. 75% of cuticle swabs yielded BFo1, but only 10% yielded BFo2, approximating the relative proportions of the two bacterial species in typical WFT (18). Frass samples contained large numbers (several thousands) of live BFo1 but relatively few BFo2 bacteria.

By day 3, BFo1 could be recovered from the vast majority of *Orius* homogenates (83%), cuticle swabs (94%) and all frass samples. In contrast, at this time point, BFo2 could not be recovered from any samples. Insects analyzed at day 10 were all F_1_ juveniles that had hatched after withdrawal of the prey WFT and from these BFo1 was recovered from 28% of homogenates, 94% of cuticle swabs and all frass samples. As for day 3, however, BFo2 could not be detected in any F_1_ generation nymphs.

The mortality rate of *O. laevigatus* was not significantly different (P>0.05) from that of the control insects over the same time period and no major change in fecundity was noted among pirate bugs predating WFT pre-infected with BFo1 and BFo2 (P>0.05). Live offspring counted at day 10 totaled 32 (from a starting population of 42 F_0_ generation individuals) compared with 37 offspring from 42 control F_0_ individuals, with the caveat that the mating status and exact age of the F_0_ individuals in each group was not determined.

## 4 Discussion

Intensive agriculture is one of several anthropogenic stressors that have led to declining insect populations (21), which in turn have had large impacts on ecosystems. This is in part due to habitat loss, but also as a consequence of increased use of indiscriminate chemical insecticides that are toxic to non-pest species. This is one driver for the development of more targeted approaches for pest insect population control. Insecticidal RNAi is a significant advance in achieving this goal.

SMR, as a means to deliver insecticidal RNAi, is of potential use to control pest insects such as WFT in which dietary dsRNA can be degraded in the upper GI tract and which are polyphagous, with potential to damage several different crop species by their feeding behavior, oviposition and by their ability to vector plant pathogenic viruses (8). Moreover, if the symbiotic bacterial species used for this purpose has a narrow host range and limited environmental persistence, the possibilities for toxic effects on non-target organisms are significantly reduced. As the first study to address this, we have compared the two principle WFT bacterial symbionts, BFo1 and BFo2.

That these bacteria have a relatively narrow host range is suggested by interrogation of insect DNA sequence databases. This analysis revealed some limited evidence for either bacterial species being present in some sequenced insect genomes. However, as the majority of these were represented by a single BioSample and very low k-*mer* counts, this indicates a likely transient association of these bacteria in insects other than WFT, in contrast to the symbiotic association with their natural host.

Excretion of both species of bacteria in the frass of WFT on plant surfaces can potentially contribute to their dissemination to other insects associated with the same plant, including pollinating species such as the European bumblebee. By combining high numbers of bacteria in an artificial bee feeding solution, we mimicked a very extreme example of environmental exposure to assess this possibility. Indeed, with these conditions, we observed that BFo1 could colonize the bumblebee GI tract, although with no significant effects on the fitness of those bees exposed to the bacteria. In contrast, even with an extended dietary exposure, BFo2 was unable to colonize the bees. It remains to be determined whether BFo1 can be transferred by colonized bees to other hive members, and if so whether this impacts overall hive fitness.

Anthocorid bugs, in particular species of the genus *Orius*, are natural predators of WFT and are widely used in WFT integrated pest management (IPM) programs (22). Consequently, these bugs can acquire both BFo1 and BFo2 bacteria when they predate on WFT. We examined the ability of both bacterial species to colonize *O. laevigatus* individually, by combining very high numbers of each separate species in an artificial feeding solution, but also in the more realistic scenario of a mixed infection *via Orius* predation on WFT. Both types of analysis revealed that whereas BFo1 could persist in and, in particular, on the cuticles of pirate bugs, even into the subsequent F_1_ generation, BFo2 failed to establish after the source of the bacteria was withdrawn from the bugs. We previously reported that BFo1 could be recovered from a pooled group of 10 individual *O. laevigatus* insects collected from a single geographical location in Spain, but was not detected in *Orius* spp. from a variety of other European field locations (23). In contrast, BFo2 was not recovered from any of these *Orius* populations.

From these analyses, we can conclude that whereas BFo1 can colonize and persist in two non-natural host insect species over several days, we found no evidence for the persistence of BFo2 in these insects. This can be attributed to the fact that, of the two species of bacteria, BFo2 is slower growing and the more fastidious, having optimal growth conditions that may mimic the WFT hindgut which it naturally colonizes (13). Moreover, we have previously demonstrated that BFo1 can suppress the growth of Gram-negative bacteria, possibly through the function of a Type VI secretion system (18), and this may contribute to its ability to establish itself in a new host. Consequently, of the two species, BFo1 is more competitive and therefore can persist in a non-natural host.

SMR can potentially contribute to integrated pest management for insects such as WFT. In terms of target specificity, SMR combines the precision of insecticidal RNAi with the host-specificity of the symbiotic bacterium. We believe that such two-tier specificity is unrivalled as a targeted insecticide, underlining the significance of our previous demonstration of SMR in thrips using BFo2 (13). Despite this bacterium being less abundant in WFT compared to BFo1, the analyses we report here vindicate the choice of the BFo2 symbiont for delivery of targeted insecticidal RNAi in WFT.

## Data availability statement

The original contributions presented in the study are included in the article/**Supplementary Material**. Further inquiries can be directed to the corresponding author.

## Author contributions

All authors contributed to the study conception and design. Material preparation, data collection and analyses were performed by MW, QX, CT, RJ, RS, MH and PD. GS and PD secured funding for the study. The manuscript was written by PD and reviewed and ultimately approved by all authors. All authors contributed to the article and approved the submitted version.
