# Supplementary material for: A narrow host-range and lack of persistence in two non-target insect species of a bacterial symbiont exploited to deliver insecticidal RNAi in Western Flower Thrips
Source: Front Insect Sci. 2023 Mar 8;3:1093970. doi: 10.3389/finsc.2023.1093970 (PMC10926499; doi:10.3389/finsc.2023.1093970)
Supplement: Supplementary file 1 [file DataSheet_1.docx]

Supplementary data

A B


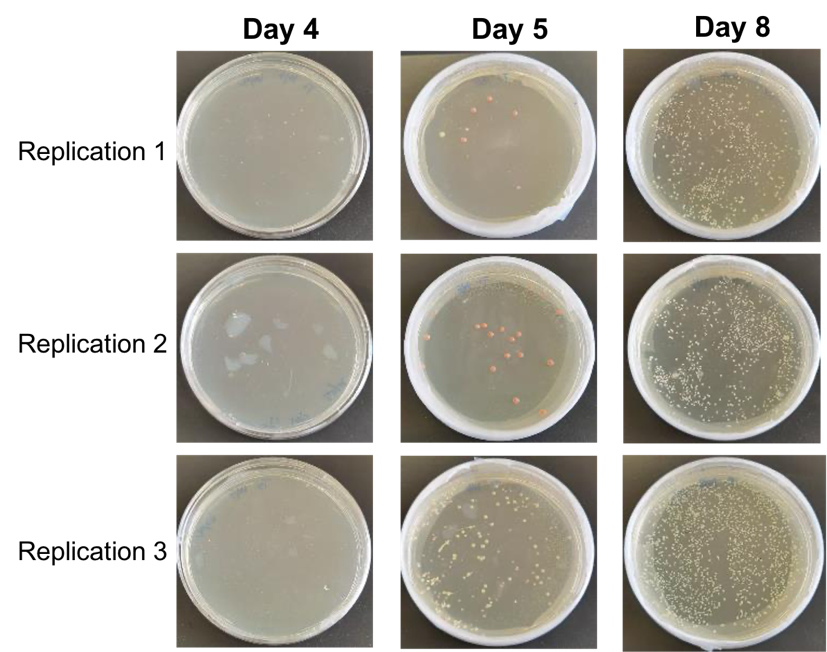

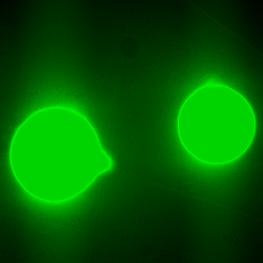


**Supplementary Fig 1:** A. Plating of bumblebee gut homogenates at different days after exposure to BFo1. B. The colonies of BFo1 on the LB plates were confirmed by fluorescence microscopy.
